# Ovarian Endometrioid Carcinoma Presenting With Presumed Right Ventricular Intracardiac Metastasis and Superimposed Thrombus: A Rare Manifestation of Gynecologic Malignancy

**DOI:** 10.7759/cureus.110452

**Published:** 2026-06-08

**Authors:** Muhammad Memon

**Affiliations:** 1 Internal Medicine, Christiana Care Health System, Newark, USA

**Keywords:** gynecologic oncology, intracardiac thrombus, intracardiac thrombus metastasis, metastatic ovarian cancer, ovarian neoplasms

## Abstract

Cardiac metastases are uncommon clinically, and intracavitary right ventricular involvement from gynecologic malignancies is exceedingly rare. We report a 69-year-old woman with multiple cardiovascular comorbidities and a recently diagnosed FIGO (International Federation of Gynecology and Obstetrics) grade 3 endometrioid carcinoma after having an exploratory laparotomy for a pelvic mass, resulting in total hysterectomy and bilateral salpingo-oophorectomy, with findings consistent with an ovarian mass and biopsy results showing endometrioid carcinoma. She was referred to the hospital after routine oncologic imaging raised concern for a right ventricular mass with associated thrombus. This patient had no symptoms on initial presentation and underwent further evaluation in the inpatient setting. Cardiac magnetic resonance imaging demonstrated a right ventricular structure extending into the right ventricular outflow tract with suspected enhancement at the attachment site to the interventricular septum, concerning for intracardiac metastatic disease with superimposed thrombus. Computed tomography of the chest demonstrated innumerable pulmonary nodules, concerning for hematogenous dissemination. Given prohibitive operative risk, surgical resection was deferred, and this was treated as presumed intracardiac metastasis with superficial thrombus. The patient was started on Warfarin for anticoagulation due to the cost-prohibitive effects of DOAC (direct oral anticoagulant) for the thrombotic component and discharged with oncology follow-up. This case highlights an exceptionally rare pattern of gynecologic malignancy involving the right ventricle, with diagnostic and therapeutic challenges related to distinguishing tumor from thrombus and balancing embolic risk against surgical morbidity.

## Introduction

Metastatic cardiac tumors are more common than primary cardiac tumors at autopsy; however, clinically recognized intracardiac metastases remain rare [1]. Gynecologic malignancies are infrequently reported as primary sources of cardiac metastases, particularly involving the right ventricle [2-5]. Primary gynecologic malignancies are usually metastatic to regional lymph nodes, such as pelvic lymph nodes, and are involved in lymphogenic spread. Lung metastasis is the most common distal organ system involved; however, cardiac metastasis is exceedingly rare [5]. Rare cases of right ventricular metastasis have been described in association with endometrial carcinoma, cervical carcinoma, ovarian germ cell tumors, and ovarian low-malignant-potential tumors. The rarity of these presentations often contributes to delayed diagnosis and challenges in clinical management [2-4,6].

Furthermore, the evaluation of intracardiac masses presents a significant diagnostic challenge. Although echocardiography is typically the first-line imaging modality, tissue characterization remains limited, and differentiation between thrombus, benign lesions, and malignant tumors can be difficult [7]. Because biopsy is often technically challenging and associated with procedural risk, diagnosis frequently relies on multimodality imaging, yet imaging findings may remain nonspecific and overlap between thrombotic and metastatic lesions, and, at times, metastatic lesions can mimic thrombus [7,8]. Essentially, for metastatic disease to the heart, CMR is the gold standard for imaging; however, findings on tissue characterization are not specific and can change based on the metastasis component [9]. We report a rare case of endometrioid endometrial carcinoma metastatic to the right ventricle that initially posed a diagnostic dilemma, underscoring the importance of maintaining a broad differential diagnosis when evaluating intracardiac masses in patients with malignancy.

## Case presentation

A 69-year-old woman with a history of type 2 diabetes mellitus, hypertension, hyperlipidemia, chronic obstructive pulmonary disease, myocardial infarction, and cerebrovascular accident presented to the outpatient setting after. She initially presented to the outpatient setting with pelvic pain and concern for a pelvic mass. She underwent laboratory testing, and it was found that she had a CA 125 of 569 U/mL, as seen in Table 1. Her malignancy was confirmed prior to admission after exploratory laparotomy, left salpingo-oophorectomy, resection of a 22-cm mass, and omentectomy. Biopsy results in the outpatient setting showed a high-grade, FIGO (International Federation of Gynecology and Obstetrics) grade 3, endometrioid carcinoma.

**Table 1:** CA 125 cancer marker drawn in the outpatient setting CA 125 = cancer antigen 125

	Patient Value	Reference Range
CA 125	569 U/mL	0-35 U/mL

Subsequently, during routine imaging for oncologic staging/surveillance, concern was raised for a right ventricular mass with associated thrombus seen on CT imaging, as seen in Figure 1. Her oncologist instructed her to present to the hospital for further evaluation. She was admitted to the hospital for further workup and evaluation. She was asymptomatic, vital signs were unremarkable, and she was found to be anemic, thrombocytopenic, and leukocytic, as seen in Table 2.

**Figure 1:**
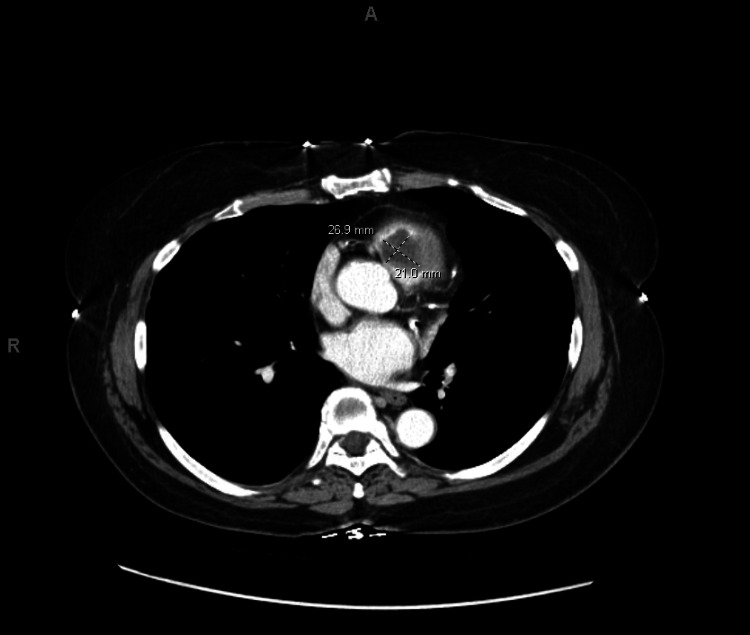
Contrast-enhanced axial computed tomography (CT) image of the chest demonstrating a 26.9 × 21.0 mm intracavitary filling defect within the right ventricle (measurement markers shown) CT = computed tomography

**Table 2:** Initial CBC on admission WBC = white blood cell; Hgb = hemoglobin; CBC = complete blood count

Test	Patient Value	Reference Range
WBC	22.2 x (10^3^/uL)	3.5 - 11 x (10^3^/uL)
Hgb	10.7 g/dL	11.7-15.7 g/dL
Platelets	116 x (10^3^/uL)	150 - 400 x (10^3^/uL)

A transthoracic echocardiogram (TTE) was done for initial evaluation, as shown in Figure 2. Cardiac magnetic resonance was then completed, which redemonstrated a right ventricular structure located in the upper aspect of the right ventricle with extension into the right ventricular outflow tract. There was suspected enhancement of the structure at the attachment site to the interventricular septum on post-contrast and subtraction sequences, concerning for an underlying mass with superimposed thrombus. In the setting of known ovarian malignancy, the finding was suspicious for intracardiac metastasis. This is seen in Figure 3.

**Figure 2:**
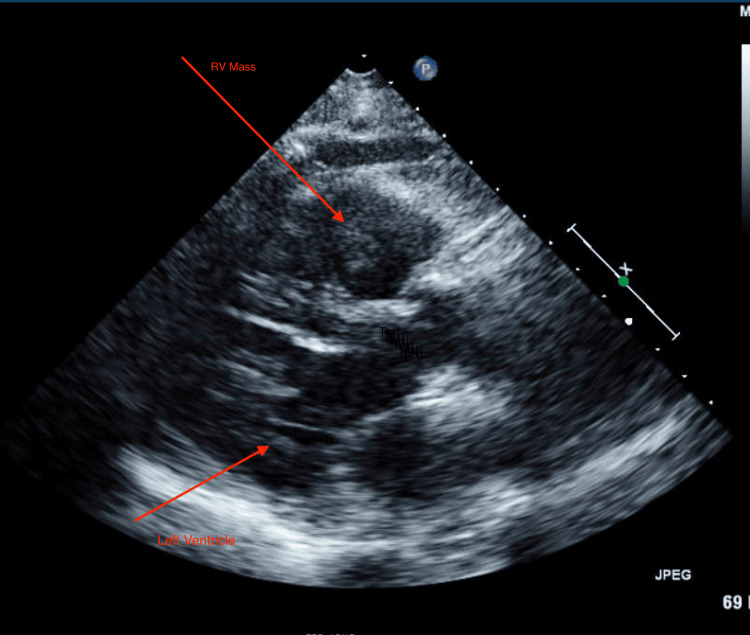
Transthoracic echocardiogram (TTE) in parasternal long-axis view showing an RV mass in the outflow tract RV = right ventricle

**Figure 3:**
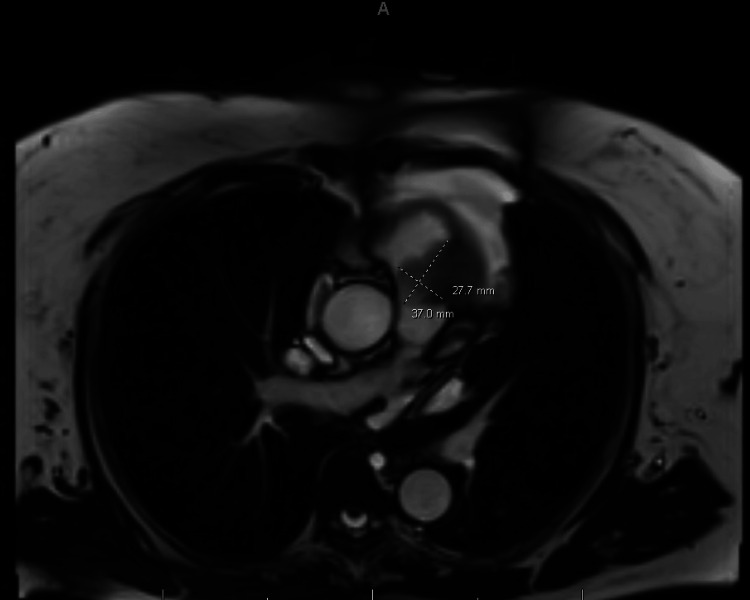
Cardiac MRI) Cine balanced steady-state free precession (bSSFP) four-chamber view demonstrating a right ventricular intracavitary mass measuring approximately 3.7 x 2.8 x 3.4 cm located in the upper right ventricle with extension into the right ventricular outflow tract. The lesion demonstrates heterogeneous signal intensity and contrast enhancement, findings more suggestive of metastatic disease than bland thrombus. MRI = magnetic resonance imaging

Computed tomography of the chest demonstrated numerous pulmonary nodules, concerning for hematogenous metastatic spread, possibly related to tumor embolization or dissemination from the right-sided intracardiac lesion.

Cardiac surgery evaluated the patient and deemed her to be at prohibitively high risk for operative removal. Given the imaging concern for superimposed thrombus, she was started on Warfarin for anticoagulation after it was found that direct oral anticoagulants (DOAC) were cost-prohibitive. She elected to be discharged with outpatient oncology and cardiology follow-up.

## Discussion

In general, distant metastatic disease of primary gynecologic malignancy is rare to begin with. The typical disease progression is usually through lymphogenic spread. We usually see spread to the regional pelvic lymph nodes, including common iliac, external, and internal iliac lymph nodes [5]. Furthermore, distal metastasis is even rarer; out of 69,027 patients studied with endometrial carcinoma, lung metastasis was deemed to be the most common; however, this was also rare, as there were only 541 patients (1.5%) to be found with this. Cardiac metastasis from a distal primary malignancy is rare, but not unprecedented. It is expected that the frequency of metastasis to “the pericardium, myocardium, great vessels, or coronary arteries is between 0.7% and 3.5% at autopsy in the general population and up to 9.1% in patients with known malignancies” [1]. However, even rarer is intracavitary metastasis, making up 3% to 5% of cardiac metastasis on autopsy [1].

Furthermore, cardiac involvement from gynecologic malignancy is rare, and right ventricular intracavitary metastasis is particularly unusual. The literature notes that although cardiac metastases may be found in 5-20% of cancer deaths at autopsy, pelvic malignancies are seldom reported as the primary source [3].

Other articles report that right ventricular metastasis from endometrioid-type endometrial adenocarcinoma rarely metastasizes to the heart, with only three cases with myocardial and pericardial spread reported antemortem at that time. Due to the lack of vascularity of the endocardium and the velocity of blood, metastasis is rarely seen [2]. Some cases report findings of intracardiac spread after patients presented with pedal edema[6].

Cardiac spread from ovarian malignancy is also rarely documented. The literature reports cardiac involvement from a recurrent ovarian tumor of low malignant potential, with tumor thrombus involving the inferior vena cava and right atrium [6]. An ovarian yolk sac tumor with a right ventricular mass and widespread hematogenous and lymphatic dissemination is also reported, describing cardiac metastasis from germ cell tumors as extremely rare, particularly in females [8]. Although these reports differ histologically from suspected ovarian endometrioid carcinoma, they demonstrate that ovarian and other gynecologic tumors can rarely involve the heart through hematogenous spread, venous extension, or tumor embolization.

The present case is notable because the right ventricular lesion demonstrated imaging features concerning both tumor and thrombus. Cardiac MRI can be useful in this setting because enhancement at the attachment site favors tumor, whereas non-enhancing components may represent thrombus. The presence of innumerable pulmonary nodules further supports hematogenous dissemination from a right-sided intracardiac source. Management is not standardized due to the rarity of the condition and must be individualized. Surgical resection may be considered in selected patients for diagnosis, symptom relief, or prevention of obstruction/embolization, but in patients with high operative risk or disseminated disease, systemic therapy and anticoagulation may be more appropriate.

## Conclusions

Right ventricular metastasis from a gynecologic malignancy is an exceptionally rare clinical entity. In this patient with ovarian endometrioid carcinoma, cardiac MRI findings suggested an enhancing right ventricular mass with superimposed thrombus, accompanied by innumerable pulmonary nodules concerning for hematogenous spread, raising suspicion of metastatic disease to the right ventricle. This case emphasizes the importance of considering intracardiac metastasis in patients with gynecologic malignancy and new cardiac masses, while recognizing that treatment decisions are often limited by surgical risk, tumor burden, and the presence of thrombus.
